# Psychometrics of the preschooler physical activity parenting practices instrument among a Latino sample

**DOI:** 10.1186/1479-5868-11-3

**Published:** 2014-01-15

**Authors:** Teresia M O’Connor, Ester Cerin, Sheryl O Hughes, Jessica Robles, Deborah I Thompson, Jason A Mendoza, Tom Baranowski, Rebecca E Lee

**Affiliations:** 1 USDA/ARS Children’s Nutrition Research Center, Department of Pediatrics, Baylor College of Medicine, 1100 Bates St, Houston, TX 77030, USA; 2 Academic General Pediatrics, Department of Pediatrics, Baylor College of Medicine, Houston, TX, USA; 3 Institute of Human Performance, The University of Hong Kong, Pokfulam Road, Pokfulam, Hong Kong SAR; 4 Centre for Physical Activity and Nutrition, School of Exercise and Nutrition Sciences, Deakin University, Burwood, Australia; 5 College of Nursing and Health Innovation, Arizona State University, Phoenix, USA; 6 Division of General Pediatrics, Department of Pediatrics, University of Washington School of Medicine, Seattle, WA, USA

**Keywords:** Physical activity, Parenting practices, Latino, Hispanic, Preschool child, Confirmatory factor analysis

## Abstract

**Background:**

Latino preschoolers (3-5 year old children) have among the highest rates of obesity. Low levels of physical activity (PA) are a risk factor for obesity. Characterizing what Latino parents do to encourage or discourage their preschooler to be physically active can help inform interventions to increase their PA. The objective was therefore to develop and assess the psychometrics of a new instrument: the Preschooler Physical Activity Parenting Practices (PPAPP) among a Latino sample, to assess parenting practices used to encourage or discourage PA among preschool-aged children.

**Methods:**

Cross-sectional study of 240 Latino parents who reported the frequency of using PA parenting practices. 95% of respondents were mothers; 42% had more than a high school education. Child mean age was 4.5 (±0.9) years (52% male). Test-retest reliability was assessed in 20%, 2 weeks later. We assessed the fit of a priori models using Confirmatory factor analyses (CFA). In a separate sub-sample (35%), preschool-aged children wore accelerometers to assess associations with their PA and PPAPP subscales.

**Results:**

The a-priori models showed poor fit to the data. A modified factor structure for encouraging PPAPP had one multiple-item scale: engagement (15 items), and two single-items (have outdoor toys; not enroll in sport-reverse coded). The final factor structure for discouraging PPAPP had 4 subscales: promote inactive transport (3 items), promote screen time (3 items), psychological control (4 items) and restricting for safety (4 items). Test-retest reliability (ICC) for the two scales ranged from 0.56-0.85. Cronbach’s alphas ranged from 0.5-0.9. Several sub-factors correlated in the expected direction with children’s objectively measured PA.

**Conclusion:**

The final models for encouraging and discouraging PPAPP had moderate to good fit, with moderate to excellent test-retest reliabilities. The PPAPP should be further evaluated to better assess its associations with children’s PA and offers a new tool for measuring PPAPP among Latino families with preschool-aged children.

## Background

Overweight and obesity among children have increased dramatically over the past decades. Latino children appear to be particularly susceptible to obesity even as preschoolers [1], and obesity-related metabolic and endocrine diseases as they get older [2]. Regular engagement in physical activity (PA) appears to reduce the risk of obesity among preschool children [3,4] particularly male preschoolers [5]. Moreover, PA may track over time in children [6], suggesting that establishing higher levels of PA in young children might be beneficial. PA for children has been defined as: “bodily movement produced by skeletal muscles that results in energy expenditure, including active play, active transportation, household chores, sports participation, and exercise” (page 142) [7] adapted from Caspersen’s definition [8]. Preschool children should get at least 60 minutes of structured PA (such as organized active play) and at least 60 minutes of unstructured PA (such as free play) daily [9].

According to Social Ecologic [10] and Social Cognitive Theories [11], PA is multi-factorial and influenced by individual, social, and physical environmental variables. For children, parents are an important social influence, influencing their PA directly, but also indirectly through children’s attraction to PA and perceived competence for PA [12]. Decades of research on parental influences on children’s behaviors support that parents are an important determinant of children’s socialization and behaviors, both through their parenting style and their parenting practices [13]. Darling and Steinberg defined parenting styles by the values and goals parents have in raising their child, the attitudes that the parents have regarding the parent-child relationship, and the parenting practices they use to attain their desired outcomes [13]. The parenting style employed is believed to establish the emotional climate between the parent and the child. Parenting practices, on the other hand, are goal oriented parenting behaviors that are specific to a context and intended to influence their child’s behavior in that context [13] (e.g. rules around homework or promoting PA). While physical activity parenting practices have been identified and linked to children’s PA among older children [14-16], few instruments have been developed to assess parenting practices in the context of PA for preschoolers.

A recent systematic review of PA parenting questionnaires [16] identified 11 available PA parenting instruments. Only one was intended for preschool-aged children [17]; and another [18], originally intended for school aged children, was used among preschoolers [16]. The review acknowledged that several measures lacked theoretical foundation, qualitative formative research, and/or appropriate validation studies, including psychometric analysis of the scales. They called for the development of comprehensive multi-dimensional PA parenting measures with appropriate validation prior to use. It is possible that the use of non-validated PA parenting scales is the reason for sometimes failing to identify associations of PA parenting practices with preschoolers’ PA in multivariate models in previous studies [19]. Other reviews have identified the lack of studies that investigated potential negative parent social support for PA that may inhibit PA or promote inactivity, and encourage investigation into this construct [20].

Among older children, parents can be an important influence on children’s PA, through active role modeling, direct involvement, encouragement, and providing transportation [21]. It is unclear if these same parenting practices are used by parents of preschool aged children or if they influence preschooler’s PA. Since cultural variables influence parenting [22] and children’s PA behaviors [23], a PA parenting practice instrument developed for use with Latino parents should be informed by qualitative studies with Latino parents. The aim of this study was therefore to a) develop a new multi-dimensional, self-report measure of Preschoolers’ PA Parenting Practices (PPAPP), and b) examine the psychometric properties (reliability, construct/factor validity and criterion validity) of the newly-developed PPAPP among a Latino sample.

## Methods

### Development of the PPAPP instrument

Darling and Steinberg [13] identified that parenting practices are not the same for all contexts and need to be operationalized for each specific context (e.g. PA parenting practices). The PPAPP instrument was therefore developed based on a qualitative formative study with Latino parents of preschool children [24], and the structure informed by current parenting paradigms [25-27] and research on parental influences on child PA [28-32].

The qualitative formative work used Nominal Group Technique (NGT) [33,34], a structured multi-step group procedure to prompt and prioritize responses from a group of people in reaction to a question or problem. Ten NGT groups (n = 74) were conducted with Latino parents who were mostly mothers. Five groups were asked to identify what Latino parents do to encourage their preschool aged child to be physically active, and 5 were asked to identify what Latino parents do that may discourage their preschool aged child to be physically active. The prioritizations of responses from the five groups who addressed the same question were aggregated into rank order lists across the groups of the PA parenting practices that encourage or discourage Latino preschool children to be physically active and have been published elsewhere [24]. We used the two prioritized lists to develop 38 items for PA parenting practices; based on parent responses in the NGT sessions the items were identified as encouraging child PA (21 items) or discouraging child PA (17 items).

**Figure 1:**
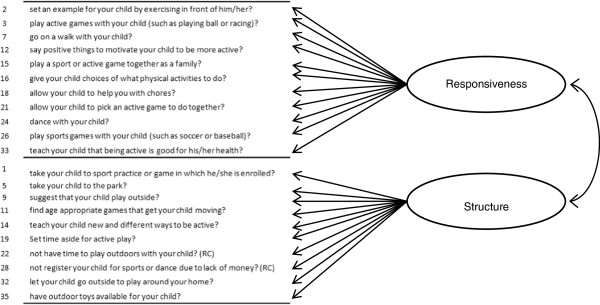
Proposed model for parenting practices that encourage Latino preschooler’s physical activity.

**Figure 2:**
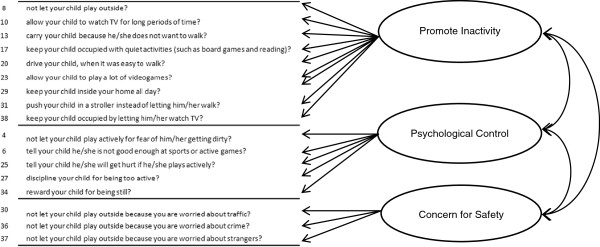
Proposed model for parenting practices that discourage Latino preschooler’s physical activity.

The items in each list were then grouped into parenting practice factors for parenting practices that Encourage PA and those that Discourage PA separately, based on constructs that have been identified for physical activity or other contexts of parenting [25-32]. The primary theoretical framework for PA parenting employed to organize the items into factors was based on a framework outlined by a working group of physical activity and parenting experts [32]. This working group [32] recommended that PA parenting practices be aligned along three previously defined dimensions of parenting: responsiveness, structure and control [25-27,35]. Parental responsiveness of PA was defined as the practices parents use to show warmth, autonomy support and reasoned communication to foster child PA individuality and self-assertion in PA [32]. Parental structure for PA was defined as how parents organize the physical environment to foster child competence in PA [32]. It was hypothesized that parenting practice items that *encouraged* child PA would measure these two factors: *responsiveness* and *structure*[32] (Figure 1). In addition, the working group suggested there may be parenting practices that negatively impact children’s PA [32], such as a control or demandingness dimension for PA. This PA dimension was defined as directive, restrictive and punitive parenting practices that force the child to be physically active. Based on this, items describing parenting practices that *discouraged* children from being physically active were hypothesized to measure three factors: *psychological control*, *restriction due to safety concerns*, and *promotion of inactivity* (Figure 2). Psychological control was defined as strategies to mold child behaviors through manipulation to satisfy the parents’ needs [25] and restriction due to safety concerns was defined as restricting activity due to concern for the child’s safety [28]. The third factor **-** promote inactivity **-** was not based on parental control, and was defined as parenting behaviors that promoted sedentary behaviors in children. This factor was added based on previous investigations by other PA parenting researchers [17,31].

Separate *a priori* reflective models [36] for parenting practices encouraging PA and discouraging PA were constructed for several reasons. First, there is some preliminary evidence that parenting practices that discourage PA may have a greater effect on preschooler’s PA than those that encourage PA, as children of this age tend to be active [37]. Second, effective and ineffective fruit and vegetable parenting practices exist as separate constructs in separate models [38,39], lending support to possible separate constructs in other parenting practice contexts. In this regard, a model of parenting practices to encourage and discourage PA based on data from this study showed that latent factors representing the two types of parenting practices were essentially independent. Third, specific practices discouraging PA may be negatively related to practices encouraging PA. Thus, a combined measurement model of parenting practices encouraging and discouraging PA would likely be overly complex with many items loading on multiple latent factors. In fact, when a combined model was tested in this study, six items loaded (in the opposite direction) onto latent factors representing both practices discouraging and encouraging PA.

The instructions for the instrument were to choose “the best answer for how often you do each of the following with your 3-5 year old child”. A 5-point Likert response scale was used (never, rarely, sometimes, often, and always). The questionnaire was first developed in English, then translated into Spanish by a staff member fluent in Spanish and English, and independently back-translated into English by a second bilingual staff to ensure content validity between the English and Spanish versions. Differences in the original and back-translated versions were reviewed and consensus was reached for conceptual and cultural rather than linguistic equivalence. Cognitive interviews [40,41] were conducted with 10 parents while completing the PPAPP instrument and other instruments in the study. Five were conducted in Spanish and 5 in English to assess parents’ understanding of the instructions, items and response options. In general, the parents correctly interpreted the PPAPP items and response options in both languages. The cognitive interviews resulted in minor rewording of four of the 38 items to enhance clarity.

### Validation study

#### Sample and recruitment

A cross-sectional study with Latino parents of preschool aged children residing in Harris County, Texas (where Houston is located) was conducted. Since neighborhood crime and traffic safety may impact children’s PA [42], this study aimed to recruit Latino parents from neighborhoods cross-stratified by these neighborhood characteristics. Crime data at the census block group level for Harris County were obtained from Tetrad Inc (Vancouver, British Columbia; http://www.tetrad.com) who modeled a crime index based on FBI Uniform Crime Report data from 1998- 2007, including personal (murder, rape, robbery, and assault) and property (burglary, larceny, and motor-vehicle thefts) crimes. Traffic-related injury data at the census block group level from 2004-2008 were obtained from the Houston-Galveston Area Council and included de-identified counts of motor vehicle, pedestrian, and cyclist accidents resulting in injuries or deaths. In addition, the motor vehicle miles traveled for each block group were estimated by the vehicle count per block group divided by the mean distance from center of block group to the border of the block group based on 2000 US census maps [43]. A principle components analysis of the traffic data revealed three factors: (1) motor vehicle injury and fatalities, (2) vehicle miles traveled and pedestrian fatalities, and (3) cyclist fatalities. A traffic safety index was calculated as the sum of the three traffic factor scores for each census block group. Based on median splits of the crime and traffic safety indices, each block group in Harris County was classified as high crime/high traffic; high crime/low traffic; low crime/high traffic; or low crime/low traffic. Recruitment attempted to get equal enrollment of participants from all four types of block groups (about 60 participants from each) and by the gender of the child.

Parents were recruited through various community organizations, events, and fliers posted in various locations; as well as the Children’s Nutrition Research Center (CNRC) website and bulletin boards, Baylor College of Medicine (BCM) website, CNRC newsletter, and phone calls to CNRC volunteer list members. Enrolled participants were asked to distribute fliers to Latino friends, relatives or neighbors with preschool aged children who may also be interested in the study. The most common ways enrolled participants reported learning about the study were fliers at clinics (36.3%), referred by friend or relative (29.2%), fliers in the community (13.3%), and contacted as part of the CNRC database (10.8%). Parents were enrolled if they met study criteria and provided written informed consent.

After informed written consent was obtained, parents were asked to complete a demographic questionnaire and self-report instruments including the new PPAPP survey. Two hundred forty one self-identified Latino or Hispanic parents completed the study, which included only one parent per household. One family withdrew during the study and their data were removed, leaving a sample of 240 for the analyses. The majority (95%) were mothers, with 3% fathers and 1% other female relative (Table 1). The average parent age was 32.3 years (±6.1), and less than half (45%) of the parents were born in the United States. Thirty percent had less than a high school education, 28% had completed high school or the equivalent, and 42% had some education beyond high school. Thirty percent reported a family income of less than $20,000, 48% from $20,000 to $49,000; and 18% more than $50,000. Over half of the sample (mostly mothers) reported not being employed currently. The average age of their preschool aged child was 4.5 (±0.9) years old and 52% reported having a preschool aged boy. The majority of the children were born in the US (99%); and 43% reported speaking mostly Spanish at home, 19% mostly English and 38% both English and Spanish. Just over half (53.3%) completed the questionnaires in Spanish and the remainder in English (Table 1).

**Table 1:** Parent and child descriptive characteristics

**Variables**	**Whole sample (*n*** **= 240)**	**Reliability sub-sample (*n*** **= 48)**	**Accelerometer sub-sample (*n*** **= 84)**
**Parent characteristics**			
Relationship to child participant, n (%)			
Mother	229 (95%)	44 (92%)	81 (96%)
Other female relative	3 (1%)	1 (2%)	3 (4%)
Father	8 (3%)	3 (6%)	0
Age, mean (SD)	32.3 (6.1)	31.5 (5.6)	32.7 (6.7)
Born in the US, n (%)	108 (45%)	22 (46%)	36 (43%)
Education, n (%)			
< High School	72 (30%)	18 (38%)	24 (29%)
High School/GED	67 (28%)	15 (31%)	22 (26%)
> High School	101 (42%)	15 (31%)	37 (44%)
Not answered	1 (<1%)	NA	1 (1%)
Current employment status, n (%)			
Not employed	129 (54%)	31 (65%)	50 (60%)
Part-time	42 (17.5%)	3 (6%)	15 (18%)
Full-time	59 (25%)	14 (29%)	16 (19%)
> 40 hr/wk	9 (4%)	n/a	2 (2%)
Not answered	1 (<1%)	n/a	1 (1%)
Total household income, n (%)			
≤ $19k	72 (30%)	14 (29%)	29 (35%)
$20k- $49K	116 (48%)	22 (46%)	37 (44%)
≥ $50k	42 (18%)	10 (21%)	13 (15%)
Unknown/No answer	10 (4%)	2 (4%)	5 (6%)
Primary language spoken in household, n (%)			
English	46 (19%)	11 (23%)	11 (13%)
Spanish	103 (43%)	22 (46%)	39 (46%)
Both	91 (38%)	15 (31%)	34 (41%)
**Child characteristics**			
Age, mean (SD)	4.5 (0.9)	4.4 (0.8)	4.4 (0.8)
Gender, n (%)			
Female	115 (48%)	20 (42%)	37 (44%)
Male	125 (52%)	28 (58%)	47 (56%)
Born in the US, n (%)	237 (99%)	48 (100%)	83 (99%)
Child physical activity (PA)			
Sedentary, min/day (SD)			369.1 (70.9)
Light PA, min/day (SD)			247.0 (36.6)
Moderate-to-vigorous PA, min/day (SD)			83.4 (38.3)
Counts/min, mean (SD)			611.8 (230.5)

Similar to other studies [44-46], a sub-sample (*n* = 48, 20% of the total sample) was asked to complete the questionnaire a second time (two weeks later) to estimate the test-retest reliability of the instrument. A second sub-sample of parents (*n* = 85, 35%), with some overlap with the first, provided additional consent to have their 3-5 year old child wear data collection monitors, including an accelerometer for a week. All participants were offered the opportunity to participate in the test-retest and objective PA assessment components of the study. Participants who expressed interest were consecutively enrolled in the test-retest subsample until the pre-set quota (20%) was met. The goal was to also stratify children by neighborhood type and gender to qualify to participate in the objective PA assessment study sub-component and consecutively enrolled until the pre-set quota (35%) was met. The two sub-samples had similar demographic characteristics (Table 1). The study was approved by the Baylor College of Medicine Institutional Review Board. Participants received $20 in compensation for completing the questionnaires, and $20 if they completed the same questionnaires two weeks later. Participants whose preschool-aged child wore the monitors for one week received an additional $30 if the parent completed a monitor wear log during that week, and the data met quality criteria described below.

### Child PA

Eighty-five children wore Actigraph GT3X accelerometers recording at 15 second epochs on their right hip [47] on an elastic fitted belt for 7 days. One family withdrew during the study, and data were removed from further analyses, leaving a sample of 84. Parents were instructed to remove the monitors at night when the child was sleeping and when the child was bathing or swimming to avoid getting the monitors wet. Parents completed a monitor wear log for their child and the non-wear time reported by the parent was removed, along with consecutive “0” counts for ≥30 minutes. The accelerometer data were considered complete if after processing, there were ≥ 480 minutes of activity data/day for at least four days, including one or more weekend days. Allowing for re-wears, 82 (96%) children had valid data. Data were processed using Pate’s cut points for preschool children [48] with 0-37 counts/15 seconds defined as sedentary, 38-419 counts/15 seconds as light PA (LPA), 420-841 counts/15 seconds as moderate PA (MPA), and ≥ 842 counts/15 seconds as vigorous PA (VPA) [48,49]. In addition, counts per minute (CPM) were used to assess overall daily activity. These cut-points have been validated with preschool children’s oxygen consumption during activities [48].

### Data analyses

Confirmatory factor analyses (CFAs) based on the Maximum Likelihood Estimation method were used to assess the fit of the data to a priori models. Jöreskog and Sörbom’s iterative model-generating approach was used to re-specify the models and was guided by an inspection of standardized factor loadings, standardized residual covariances, univariate Langrage multiplier tests, Wald tests, multivariate outliers, and theoretical considerations [50]. Global model fit was tested using the comparative fit index (CFI), the root mean square error of approximation (RMSEA), and the standardized root mean squared residual (SRMR) [51,52]. According to Hu and Bentler [51], values supportive of good model fit are ≥0.95 for CFI, ≤0.06 for RMSEA, and ≤0.08 for SRMR. Given that the CFI is sensitive to the magnitude of correlations between variables [52], we treated CFI values ≥0.90 as indicative of acceptable levels of model fit if the other two fit indices met Hu and Bentler’s stricter criteria. We also reported the Satorra-Bentler scaled χ^2^ test (robust to violation to the normality of distribution assumption) [53]. The following parameters were used to examine local fit of the models: standardized factor loadings, standardized residual covariances, univariate Langrage multiplier tests and Wald tests. Eqs 6.2 (Multivariate Software Inc., Encino, CA, 2010) was used to conduct CFAs.

Intra-class correlation coefficients (ICCs) were computed to establish the test-retest reliability of the PPAPP subscales and single items derived from the final measurement models. ICCs were computed using absolute-agreement in two-way random models [54]. ICC values up to .20 denote poor reliability; .21-.40 fair reliability; .41-.60 moderate reliability; .61-.80 substantial level of reliability; and > .80 excellent reliability [55]. Cronbach’s alpha coefficients and mean inter-item correlation were computed to establish the internal consistency of the subscales in the total sample. Cronbach’s alphas were also calculated separately for instruments completed in English and Spanish. Mean inter-item correlations were also calculated as a more straightforward assessment of internal consistency that is not item number sensitive, with values ranging from .15-.50 considered indicative of an adequate level of internal consistency [56].

Associations of PA parenting practice subscales/items with child PA were assessed using hierarchical regression analyses whereby accelerometer wear-time, child’s gender and child’s age (potential confounders) were entered in the regression models in a first step, and a specific parenting practice sub-scale/item was entered in a second step. The resulting increase in percent variance explained (R^2^) of PA after adding a parenting practice subscale/item was square-rooted to obtain a measure of confounder-adjusted measure of association between a specific PA measure and the parenting practice subscale/item. As daily minutes of vigorous PA was non-normally distributed (positively skewed), they were log-transformed for the purpose of these analyses. Significance was set at *p* < .10 given the exploratory nature of these last analyses in a relatively small sub-sample [57].

## Results

### Confirmatory factor analyses

**Figure 3:**
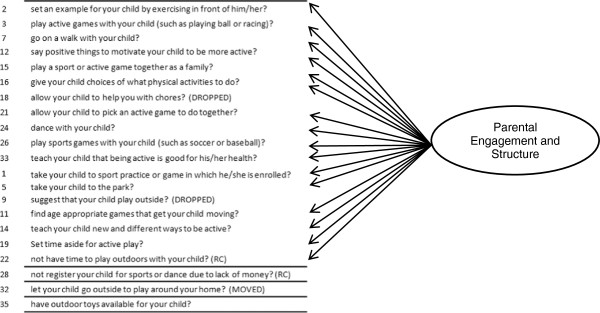
Final model for parenting practices that encourage Latino preschooler’s physical activity.

**Figure 4:**
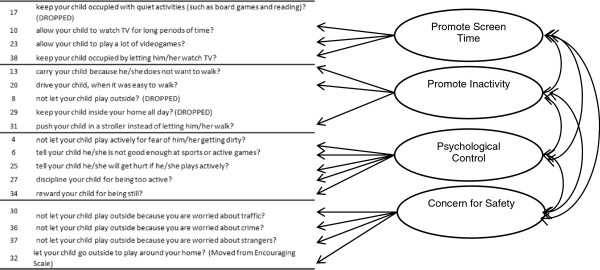
Final model for parenting practices that discourage Latino preschooler’s physical activity.

The *a priori* factor structure of the PPAPP subscales for Encouragement and Discouragement of PA demonstrated poor fit to the data, with none of the model-fit indices meeting the pre-established criteria (Table 2). The models were re-specified to improve the fit to the data but retain their conceptual structure (Figure 3). The measurement model for the PPAPP – Encouragement scale was modified by first excluding items with low communalities (defined as standardized loadings < |.30|) [58] that were not significantly correlated with children’s objectively-measured PA (3 items). An additional item (let your child go outside to play around your home) was moved to the PPAPP - Discouragement scale as it had low communality and was conceptually and empirically related to several items gauging Restriction for Safety Concerns, a latent factor underlying responses on the PPAPP – Discouragement scale (see Tables 3 &4; Figures 3 &4). Although, two single items [not register child for sport/dance due to lack of money (reverse coded, such that higher score approaches “never”); and have outdoor toys available for child] had low communalities (Table 3), they were retained because they significantly correlated with children’s PA in the sub-sample (Table 5). Given that the PPAPP-Encouragement latent factors Parental Responsiveness and Structure were highly correlated (*r* = 0.95), they were combined into a single Parental Engagement factor. The final measurement model for PPAPP - Encouragement (Table 3; Figure 3), consisting of one latent factor (parental engagement: 15 items) with two single items, demonstrated acceptable fit to the data with two indices meeting Hu and Bentler’s [51] stricter criteria of model fit (Table 3).

**Table 2:** **Fit indices of a *priori *** **of measurement models of parenting practices that encouraged or discouraged physical activity (PA) among Latino preschool children**

**Model fit indices *(Poor Fitting Models)***			
**Parenting practices encouraging PA**		**Parenting practices discouraging PA**	
Satorra-Bentler scaled χ^2^ (df = 85)	393.2; *p* < .001	Satorra-Bentler scaled χ^2^ (df = 74)	199.7; *p* < .001
CFI	0.86	CFI	0.88
RMSEA (95% CI)	0.07 (0.06, 0.07)	RMSEA (95% CI)	0.06 (0.03, 0.08)
SRMR	0.08	SRMR	0.07

**Table 3:** Final measurement model of parenting practices that encourage Latino preschool children to be physically active and item characteristics (mean; standard deviation; skewness; corrected item-scale correlation: standardized loading)

**Items**	**Parental engagement**			
**How often do you…**	***M *** **( *SD *)**	**Skewness**	**CISC**	**Standardized loading**
Set an example for your child by exercising in front of him/her?	3.11 (1.14)	0.09	0.66	0.67
Play active games with your child (such as playing ball or racing)?	3.44 (0.94)	0.08	0.71	0.75
Go on a walk with your child?	3.36 (0.93)	0.03	0.55	0.59
Say positive things to motivate your child to be more active?	4.25 (0.82)	-0.82	0.56	0.56
Play a sport or active game together as a family?	3.25 (0.93)	0.05	0.69	0.75
Give your child choices of what physical activities to do?	3.53 (0.87)	0.03	0.62	0.69
Allow your child to pick an active game to do together?	3.39 (0.88)	0.20	0.63	0.68
Dance with your child?	3.54 (0.90)	0.03	0.47	0.49
Play sports games with your child (such as soccer or baseball)?	2.91 (1.00)	0.11	0.58	0.61
Teach your child that being active is good for his/her health?	3.28 (1.10)	-0.49	0.61	0.64
Take your child to the park?	3.41 (0.82)	0.26	0.45	0.52
Teach your child new and different ways to be active?	3.47 (0.92)	0.16	0.77	0.84
Take your child to sport practice or game in which he/she is enrolled?	2.28 (1.45)	0.66	0.37	0.31
Find age appropriate games that get your child moving?	3.49 (0.89)	-0.17	0.49	0.53
Set time aside for active play?	3.10 (1.05)	-0.01	0.65	0.67
**Retained single items**				
Not register your child for sports or dance due to lack of money? (RC)	3.03 (1.43)	NA	-0.02	0.00
Have outdoor toys available for your child?	3.88 (1.22)	NA	-0.84	0.00
**Dropped items**				
Allow your child to help you with chores?	3.65 (0.95)	NA	-0.31	
Suggest that your child play outside?	3.22 (1.07)	NA	-0.03	
Not have time to play outdoors with your child?	2.97 (0.96)	NA	0.38	
Let your child go outside to play around your home? (moved to discourage)	3.30 (1.10)	NA	-0.51	
**Model fit indices *(Moderately-fitting model)***				
Satorra-Bentler scaled χ^2^ (df = 116)	240.94	*p* < .001		
CFI	0.90			
RMSEA (95% CI)	0.06	(0.05, 0.07)		
SRMR	0.08			

**Legend:** RC = reverse coded; NA = not applicable; M = mean; SD = standard deviation; CISC = corrected item-scale correlations.

**Table 4:** Final measurement model of parenting practices that discourage Latino preschool children to be physically active and item characteristics (mean; standard deviation; skewness; corrected item-scale correlation; standardized loading)

**Items**	**Promote screen time**				**Promote inactivity**				**Psychological control**				**Restriction for safety concern**			
***How often do you…***	**M (SD)**	**Sk**	**CISC**	**SL**	**M (SD)**	**Sk**	**CISC**	**SL**	**M (SD)**	**Sk**	**CISC**	**SL**	**M (SD)**	**Sk**	**CISC**	**SL**
Allow your child to watch TV for long periods of time?	2.64 (0.91)	0.34	0.53	0.94												
Allow your child to play a lot of videogames?	1.92 (0.90)	0.73	0.32	0.31												
Keep your child occupied by letting him/her watch TV?	2.63 (0.85)	0.13	0.45	0.55												
Carry your child because he/she does not want to walk?					2.02 (0.96)	0.74	0.35	0.94								
Drive your child, when it was easy to walk?					2.66 (1.19)	0.32	0.28	0.45								
Push your child in a stroller instead of letting him/her walk?					1.45 (0.85)	2.13	0.33	0.46								
Not let your child play actively for fear of him/her getting dirty?									1.81 (1.01)	1.23	0.29	0.41				
Tell your child he/she is not good enough at sports or active games?									1.33 (1.00)	2.93	0.22	0.31				
Tell your child he/she will get hurt if he/she plays actively?									2.40 (1.13)	0.54	0.46	0.56				
Discipline your child for being too active?									1.90 (1.02)	0.81	0.34	0.51				
Reward your child for being still?									2.42 (1.15)	0.42	0.40	0.61				
Not let your child play outside because you are worried about traffic?													2.72 (1.20)	0.31	0.56	0.58
Not let your child play outside because you are worried about crime?													2.53 (1.13)	0.46	0.73	0.93
Not let your child play outside because you are worried about strangers?													2.65 (1.09)	0.41	0.74	0.92
Let your child go outside to play around your home?													3.30 (1.10)	0.51	0.49	-0.48
***Dropped items***																
Not let your child play outside	2.63 (1.07)	0.36														
Keep your child occupied with quiet activities (such as board games and reading)?	3,16 (0.93)	0.15														
Keep your child inside your home all day?	2.58 (0.97)	0.17														
**Model fit indices *(Well fitting model)***																
Satorra-Bentler scaled χ^2^ (df = 88)						114.67			*p* < .05							
CFI						0.96										
RMSEA (95% CI)						0.04			(.01, .05)							
SRMR						0.06										

**Legend:** M = mean; SD = standard deviation; Sk = skewness; CISC = corrected item-scale correlations; SL = standardized loading.

**Table 5:** Associations of parenting practices subscale scores with physical activity variables (partial correlation coefficients)

**Scale/item**	**Sedentary**	**Light PA**	**Moderate PA**	**Vigorous PA**^**#**^	**MVPA**	**Average CPM**
Parental engagement (LA-PAPP- Encouragement)	0.03	-0.06	0.03	0.03	0.03	0.00
Not register your child for sports or dance due to lack of money (LA-PAPP- Encouragement; reverse scored)	-0.15α	0.21*	0.18*	0.08	0.09	0.11
Have outdoor toys available for child (LA-PAPP- Encouragement)	-0.09	0.05	0.13	0.16 α	0.13	0.15 α
Safety concerns (LA-PAPP- Discouragement)	0.03	-0.04	0.00	-0.05	-0.03	-0.06
Promote inactivity (LA-PAPP- Discouragement)	0.15α	-0.18α	-0.14	-0.08	-0.09	-0.12
Promote screen time (LA-PAPP- Discouragement)	0.23**	-0.28**	-0.23*	-0.15	-0.20α	-0.20α
Psychological control (LA-PAPP- Discouragement)	-0.15α	0.15	0.21*	0.05	0.13	0.13

**Legend:** PA = physical activity; MVPA = moderate-to-vigorous physical activity; CPM = counts per minute # = positively skewed variable log-transformed before estimating the associations.

All associations adjusted for wear-time, child’s gender and child’s age.

α p < .10; * p < .05; ** p < .01.

The measurement model of the PPAPP – Discouragement scale was re-specified by deleting three items with low communalities, defined as < |.30|, (all of which belonged to the Promote Inactivity sub-scale), and splitting the Promote Inactivity latent factors into two latent factors: Promote Inactivity and Promote Screen Time (Table 4; Figure 4). This resulted in four latent factors: *Promote Screen Time* (3 items), *Promote Inactivity* (3 items), *Psychological Control* (5 items), and *Restriction for Safety Concerns* (4 items). Promote Inactivity was positively related to Promote Screen Time (r = .27) and Psychological Control (*r* = .66). The final model fit the data well, with all indices meeting Hu and Bentler’s criteria [51] (Table 4).

### Scale reliabilities

The ICCs of the subscales from the test-retest sample (*n* = 48) indicated moderate to excellent test-retest reliabilities ranging from 0.56 for Restriction for Safety Concerns to 0.85 for Parental Engagement and Psychological Control (Table 6). The internal reliability of the Parental Encouragement subscale for the total sample was excellent (Cronbach’s α = 0.90; average inter-item correlation 0.38), but less consistent for the PPAPP- Discouragement subscales with fewer items. The average inter-item correlation, a more appropriate assessment of internal reliability in scales with less than 5 items [59] was acceptable for all four Discourage subscales (>.15) [56]. Some of the Discouraging PA sub-scales also had lower Cronbach’s α if the questionnaire was completed in Spanish (Table 6). However, post-hoc analyses showed that educational attainment may be confounding this finding, with participants who completed the questionnaire in Spanish less likely to have completed secondary education than their counterparts (p < 0.001, data not shown).

**Table 6:** Subscale test-retest and internal reliabilities

	**Test-retest reliability (n = 48)**			**Internal reliability (n = 240)**			
**Subscales**	**ICC (95% CI)**	**Mean (SD)**		**Average inter-item correlation**	**Cronbach’s alpha**		
		**Assessment 1**	**Assessment 2**		**Total**	**English**	**Spanish**
**Practices that encourage child PA**							
Engagement (15 items)	0.85 (0.75, 0.91)	3.51 (0.62)	3.47 (0.58)	0.38	0.90	0.87	0.92
How often do you not register your child for sports or dance due to lack of money? (single item)	0.62 (0.41, 0.76)	2.81 (1.42)	2.52 (1.22)				
How often do you have outdoor toys available for your child? (single item)	0.57 (0.34, 0.74)	3.69 (1.37)	3.77 (1.24)				
**Practices that discourage child PA**							
Promote inactivity (3 items)	0.59 (0.38, 0.85)	2.09 (0.80)	1.92 (0.70)	0.26	0.50	0.53	0.43
Promote screen time (3 items)	0.62 (0.41, 0.77)	2.30 (0.65)	2.23 (0.56)	0.34	0.61	0.65	0.57
Psychological control (5 items)	0.85 (0.75, 0.91)	2.11 (0.70)	2.03 (0.69)	0.26	0.59	0.67	0.49
Restriction for safety concerns (4 items)	0.56 (0.33, 0.73)	2.62 (0.88)	2.55 (0.95)	0.53	0.82	0.81	0.80

**Legend:** ICC = Intra-class correlation.

Assessment 1 and 2 were about 2 weeks apart.

### Correlation of PA parenting practice subscales with child PA

Several of the parenting practice subscale scores had significant (*p* < .10) correlations with children’s objectively measured PA (Table 5). Promote inactivity was negatively associated with children’s LPA and positively with sedentary time; and promote screen time negatively correlated with children’s LPA, MPA, and CPM; and positively correlated with children’s sedentary time (Table 5). While parental engagement was not correlated with children’s PA, the single items “Not register your child for sports or dance due to lack of money”, (reverse coded) was positively correlated with children’s LPA and MPA, and negatively correlated with children’s sedentary time; and “Have outdoors toys available for child”, was positively correlated with VPA and CPM. Psychological control was negatively related to sedentary time and positively related to MPA.

## Discussion

Given the prevalence of obesity among Latino children [1], and the protective effects of PA on obesity [4] and other health outcomes [60,61], understanding how parents influence their children’s PA is critical to promote healthy lifestyles among families. Valid and reliable measures of PA parenting practices are needed to fully investigate such influences and assess the effects of family based PA interventions. This study demonstrated moderate-to-good factorial validity, moderate-to-excellent test-retest reliabilities, and acceptable internal consistency reliabilities of a new parent-report instrument with two independent scales for parenting practices that influence preschooler’s PA: the Encouraging and Discouraging PPAPP. One of the scales included parenting practices that encourage Latino preschool-aged children to be physically active. The other scale included parenting practices that discourage them from being physically active comprised of four factors: promote screen time, promote inactivity, psychological control, and restriction for safety concerns.

Although the existing literature on PA parenting practices has focused on practices intended to positively impact mostly older children’s PA [21,62], this study demonstrated that among Latino preschool children, parenting practices can also discourage PA, as indicated by correlations of both the Promote Inactivity (p < .10) and the Promote Screen Time (p < .01) subscales with children’s objectively measured PA. Gubbels et al. have previously demonstrated that, among a Dutch sample of 5 year olds, parental restriction of sedentary time (measured by a 6-item non-validated subscale) was associated with parent reports of lower child PA [17]. However, to our knowledge this is the first PA parenting scale that has included a subscale to assess whether parents promoted inactive transport among children, such as pushing them in a stroller or carrying them when they could have walked. Active transportation has been associated with adolescents’, but not children’s, PA [63]. The initial exploratory analyses presented here suggest that parenting to promote inactive transport may be associated with less LPA among Latino preschoolers and warrants further investigation.

Also new in this PA parenting scale is the construct of psychological control by parents which may undermine or inhibit children’s PA. The construct of psychological control has been dominant in the developmental psychology parenting literature for decades [25] and suggested for PA parenting [32], but has not been assessed in the PA parenting literature. For this instrument, psychological control was created based on qualitative research with Latino parents who reported they believed that parents who criticize or insult their child, discipline their child for being active, or restrict their child’s activities for fear of them getting hurt tend to discourage their child from engaging in PA [24]. It was found to be positively associated with Latino preschooler’s moderate PA and negatively associated with their sedentary time. In this cross-sectional study we cannot delineate whether psychological control causes young children to be more active, or whether parents whose children are more active are more likely to use psychological control.

The fourth sub-scale for Discouraging PPAPP was restriction for safety concerns. A systematic review demonstrated that neighborhood safety was associated with children’s PA [42]; and it is likely that this relationship is in part mediated by parents’ concerns about the safety of the environment in which they allow their child to play. The new subscale of Restriction for Safety Concerns will allow further exploration of this hypothesis. Future research in larger and/or longitudinal samples will need to identify whether these constructs are important social determinants of children’s PA.

The discouraging subscales had slightly lower mean scores compared to the encouraging scale or single items (Table 6), which may suggest that parents are less likely to use parenting practices that discourage PA, but when used they have an important negative impact on children’s PA. Alternatively, parents may be less likely to self-report using parenting practices that discourage their child from being active.

Parenting practices that encourage child PA included one subscale for parental engagement for child PA that represented parenting practices that are responsive and provide structure to support PA [32]. Previous work in developmental psychology has demonstrated that parental involvement or engagement in children’s schooling mediated the positive effects of authoritative parenting on children’s school success [26]. Similarly, parental engagement or involvement with young children’s PA may positively influence children to be physically active. The addition of two single items to the encouraging PA parenting practice scale suggests that these two items represent additional latent constructs that warrant further development with additional items. These two items [not register child for sport/dance due to lack of money (reverse coded); and have outdoor toys available for child] may be markers of parenting practices as well as living conditions, such as family income and/or type of residence. As such they may not represent actual parenting practices, but rather socio-economic factors that influence parenting behaviors.

This study has many strengths: the systemic development of the PA parenting practice scales were based on a proposed theoretical framework for PA parenting [32], qualitative studies with Latino parents of preschoolers [24]; extensive psychometric analyses of the new PPAPP scales were conducted; and the criterion validity was based on objectively-measured child PA. However, there are also limitations. The PPAPP is a self-report instrument and is therefore prone to reporting biases, including socially desirable responses. However, self-report instruments are needed to investigate the influence of parents on children’s PA in larger samples, since direct observations are costly and often impractical. Additional studies to assess the convergent validity of this instrument with parent behaviors using direct observations would help strengthen its validity. The parents completed the PPAPP in English or Spanish, but the sample size was not large enough to look at the factorial invariance separately for the two languages. Instead, Cronbach’s alphas were assessed separately for those who completed the questionnaire in English or Spanish. Some variations in Cronbach’s alphas were found with lower scores among some of the Spanish language sub-scales for Discouraging PA. It is possible this is due to differences in the robustness of those subscales by language. However, educational attainment was significantly lower among those who completed the questionnaire in Spanish than those who completed it in English. Participants with low educational attainment may have found it more difficult to respond to some of the items. Among older samples of children, mothers and fathers have different influences on their children’s PA [14,20], suggesting their influence should be assessed separately. However, the scales should be developed for use with both mothers and fathers, which is why the samples were combined in this developmental study. Only a subsample of children wore the accelerometers which limited our ability to detect correlations of parenting practices subscales with children’s PA.

Future research should assess the predictive validity of the PPAPP on Latino preschool children’s PA in larger samples and the potential different influence by mothers and fathers on their same and different gendered children [14,20]. In addition, the PPAPP instrument should be refined by expanding the single items in the Encourage PA scale to multiple-item subscale. The psychometrics and predictive validity of this scale should also be cross-validated in another Latino sample and assessed in other populations, such as Non-Hispanic white, African American, and Asian, to assess its usefulness for assessing PA parenting practices regardless of race or ethnicity.

## Conclusion

The PPAPP had moderate-to-good factorial validity, moderate-to-excellent test-retest reliabilities, and acceptable internal reliabilities among a sample of Latino parents of preschool children. This new and preliminary instrument should be further evaluated to assess its association with children’s PA and offers a new research tool for measuring parenting practices among Latino families.

## Abbreviations

BCM: Baylor College of Medicine; CFA: Comparative fit analysis; CFI: Comparative fit index; CNRC: Children’s Nutrition Research Center; CPM: Counts per minute; ICC: Intra-class correlation coefficient; LPA: Light physical activity; MPA: Moderate physical activity; NGT: Nominal group technique; PA: Physical activity; PPAPP: Preschoolers’ PA Parenting Practices; RMSEA: Root mean square error of approximation; SRMR: Standardized root mean-square residual; VPA: Vigorous physical activity.

## Competing interests

The authors declare they have no competing interests.

## Authors’ contributions

TMO was the PI on the NIH funded R21 study: Ninos Activos, developed the study design for the development of the physical activity parenting practice questionnaire, oversaw all aspects of the study protocols, data collection, and drafted the manuscript. EC developed the Ninos Activos recruitment strategies and plans for analyses, conducted the analyses for this study, and regularly attended study meetings. SOH, a parenting expert in child feeding, assisted with the qualitative formative studies to inform the PA parenting items, provided expertise in expected factor structure of the PA parenting scales, regularly attended study meetings, and helped interpret the data analyses. JR was the study coordinator on the project who recruited and coordinated informed consent, and data collection of all participants. She regularly attended study meetings, and provided critical input on implementing study protocols. DIT is a qualitative researcher who trained the study staff in cognitive interview methodology, and informed the development of the interview questions. JAM provided input on the Ninos Activos study design and interpretation of analysis during bi-weekly study meetings. TB provided input on the Ninos Activos study design and interpretation of analysis during bi-weekly study meetings. REL provided input on the Ninos Activos study design and interpretation of analysis during bi-weekly study meetings. All authors critically read, edited, and approved the manuscript.
